# Can Team-Based Learning (TBL) Be Used to Deliver Postgraduate Education in Transfusion Medicine for UK Physicians?

**DOI:** 10.1007/s40670-019-00844-9

**Published:** 2019-12-03

**Authors:** Jane Graham, Conrad Hayes, Kate Pendry

**Affiliations:** 1 Department of Clinical Haematology, University Hospitals of North Midlands NHS Trust, Royal Stoke University Hospital, Newcastle Road, Stoke-on-Trent, Staffordshire ST4 6QG UK; 2 School of Medicine, Keele University, Staffordshire, UK; 3 Patient services, NHS Blood & Transplant, Manchester University NHS Foundation Trust, Manchester, UK

**Keywords:** Teaching methods: team-based learning, Flipped-classroom, Clinical teaching: residents, Postgraduate education, Graduate medical education, Transfusion medicine, Red cell prescribing

## Abstract

**Background:**

There is global need for evidence-based methodologies to effectively deliver transfusion training. This research critically assesses both efficacy and the practicalities of introducing team-based learning (TBL) to deliver transfusion medicine education to UK postgraduate doctors (residency equivalence).

**Study Design and Methods:**

One TBL orientation session and three transfusion medicine sessions, mapped to the 2012 Foundation Programme curriculum, were designed adhering to TBL principles. These were delivered by one tutor during ‘compulsory’ (except rota commitments and leave) educational sessions. Team continuity plus trainee reaction, knowledge acquisition and behaviour were evaluated.

**Results:**

Forty-eight doctors received a mean 2.5 TBL sessions. Five teams were developed with average team membership of 5.85 doctors per session. Overall team continuity (total team members attending/potential team members × 100) was 65% over the four sessions. Qualitative and quantitative trainee reaction to TBL was positive. Objective knowledge acquisition showed improved team knowledge over individual knowledge. Mean team readiness assurance testing (RAT) score exceeded maximum individual RAT score in 90% of cases. Subjective knowledge acquisition significantly improved, although confidence concerning prescribing declined. The reported time spent preparing for sessions correlated with enjoyment, subjective knowledge gain and clinical confidence. Preparation time was reported as ‘adequate’ or ‘excellent’ in 86% of anonymous feedback.

**Conclusion:**

TBL is an enjoyable and effective approach to deliver transfusion education to doctors, particularly when preparation is adequate. Team continuity is poor despite ‘compulsory’ education sessions. This must be considered when designing and delivering TBL sessions in the UK postgraduate medical setting.

## Introduction

Education and competency assessment within transfusion medicine remain key issues in patient safety. Adverse events in transfusion most likely occur because of human error rather than pathological reaction [1]. Current educational methods fail to equip doctors with adequate knowledge [2–4] or clinical competency [5], and potentially avoidable errors often involve junior medical staff in the decision making or prescribing of blood products [1]. Current methods of delivering transfusion education don’t work [6, 7] and so we need new methods to effectively equip postgraduate doctors with the required competencies to enable safe transfusion practice [7].

Team-based learning (TBL) is a form of flipped-classroom, co-operative learning [8], whose principles are summarised in Text box 1. TBL has benefits both for the learner and faculty; helping trainees develop auxiliary skills to knowledge acquisition such as interpersonal and team working skills, and enabling the faculty to deliver small group teaching to many trainees within one room, facilitated by a single expert. TBL has been widely adopted in the USA for undergraduate medical education and despite a slow start in the UK [9], this methodology is now establishing itself across a variety of health professions (www.teambasedlearning.org). Within the postgraduate/graduate medical setting there is limited evidence that TBL is feasible, however, the organisational context, learner characteristics, development of objectives/assessments and underpinning structure of effective TBL is not well described in the literature [10].

**Table:** Text box 1 Outline of team-based learning (TBL)

**Pre-course preparation**—including learning outcomes and variety of learning methods **Formation of teams**—allocated teams of 5–7 learners ensuring maximum diversity **Individual Readiness Assurance Test** (iRAT)—assesses individual foundation knowledge **Team Readiness Assurance Testing** (tRAT)—assesses group knowledge and team working skills. Immediate feedback provides additional formative learning **Instructor clarification**—clarifies concepts/learner questions **Team Application** (tAPP)—assesses team application of knowledge/skills to real-life significant problems to consolidate learning. Requires team debate and discussion to agree a specific action **Instructor clarification**—inter-team debate with instructor clarification where necessary **Peer evaluation**—provide anonymous individual feedback for team members to establish team accountability and reinforce positive behaviours concerning non-technical skills	

Dedicated postgraduate training in the UK (residency equivalence) has always been labelled ‘compulsory’, however it was recognised that service delivery often took priority over education. To improve attendance at educational sessions, the past 5–10 years have therefore seen teaching move away from 1–4-h sessions delivered during the working day, to standalone educational days totally removed from clinical practice; with attendance ‘mandated’ unless the doctor is on nights, on call or on annual leave.

Evaluation of postgraduate educational programmes is a necessary part of quality management within the UK, with Local Education Providers feeding into the national Quality Improvement Framework [11–13]. The ability to evaluate progression against learning outcomes is integral to curriculum design and educational tool development. Evaluation should consider the view of stakeholders, including trainees, faculty and any organisation that has a vested interest in the educational tool or outcome. In addition, evaluation should assess the acquisition of additional skills such as leadership, communication and team working that occur during the learning process.

For the past 50 years Kirkpatrick’s four levels of learning evaluation has been employed widely across the globe to evaluate training and learning [14]; evaluating reaction, learning, behaviour and results, either alone or in combination. This study uses Kirkpatrick’s levels of learning to evaluate the effectiveness of TBL to deliver transfusion medicine training to UK postgraduate doctors. By carefully describing implementation of TBL according to Haidet et al.’s seven core elements [15], we hope meaningful conclusions can be drawn by others concerning the use of TBL within the postgraduate medical setting, whilst simultaneously supporting the development of evidence-based pedagogy in transfusion medicine.

## Method

### TBL Intervention

Transfusion education was delivered to a cohort of first year (F1) postgraduate doctors (*n* = 48) working within a single hospital. Four sessions of TBL were delivered during ‘compulsory’ weekly teaching; attended by all F1 doctors unless on nights, on call or on leave. A single 1-h TBL orientation session (designed by JG) was followed 3 weeks later by three consecutive 2 hour weekly sessions dedicated to transfusion medicine. Session 1 covered ‘Indications for transfusion’, session 2 ‘Acute Transfusion Reactions (ATRs)’ and session 3 ‘Haemostasis & thrombosis’. The TBL transfusion sessions were designed by a faculty of transfusion medicine experts and educationalists, mapped to the 2012 Foundation Curriculum [16]. All sessions were delivered by the lead author. None of the trainees reported previous experience of TBL. The research was approved by Pennine acute hospitals NHS trust (15RECNA05). No ethical approval was required. Informed consent was obtained from all individual participants included in the study.

Teams of 5–7 persons were formed by random allocation by the tutor during the first session, as the trainees were uniform in clinical training and experience. Trainees remained in their allocated group for all four sessions. Additional trainees not present at session 1 were randomly allocated to existing groups to maintain a minimum group size of 5. Preparatory material for each session was emailed to all F1 doctors by the postgraduate education coordinator 6 days prior to each session and a suggested time of 1–2 hours was recommended for participants to spend on the pre-course material. Text length was maximum 1200 words (4 sides) detailing session learning objectives, core knowledge and links to online reading material and videos (length 2–11 min). Each 2-hour session began with an introduction to the session’s learning objectives, plus an outline of TBL for those who had not attended a previous session.

Closed-book readiness assurance testing (RAT) was delivered as 10 best-of-five questions to individuals (iRAT) and then again within teams (tRAT). Named iRAT score sheets were handed in and later scored by the tutor, with 10 points for each correct answer. Immediate feedback was provided to teams during tRAT administration using immediate feedback-assessment technique (IF-AT) forms, obtained from Epstein Educational Enterprises (www.epsteineducation.com). Ten points were scored for 1st answer correct, 6 for 2nd, 2 for 3rd and 1 for 4th. In-class problem solving was encouraged through two team application cases (tAPPs) each involving 10 minutes intra-team discussion followed by 15 minutes inter-team discussion. All teams worked on the same, significant clinical problem at the same time to make specific choices concerning investigation, management and/or diagnosis. Answers were specific but free text to better represent real-life clinical practice. Responses were written down by teams to ensure they committed to their answer. A member of each team, picked at random by the tutor from a team list, fed back to the entire group the rationale behind their teams’ decision. Discussion and debate between the groups was guided by the tutor. Reporting was simultaneous through documentation on flipcharts, with the correct answer presented by the tutor and opportunities for verbal appeal. In this study tAPP cases were used for formative assessment and learning, so were not scored in this study.

Incentives were provided by acknowledging the team with the highest tRAT score after each session and total tRAT score after completion of the course. Peer review of team performance was performed on course completion with trainees completing individual questionnaires. These used quantitative and qualitative tools to address team member participation and self-evaluation (appendix 1).

### Outcome Measures

Learner reaction to TBL was assessed in a variety of ways to include both quantitative and qualitative assessment. Trainees were provided with slips of paper by the tutor at the end of each TBL session and asked to provide anonymous qualitative feedback, specifically something positive about the session and something that could be improved.

Objective assessment of knowledge acquisition was assessed through analysis of iRAT and tRAT scores for each session. Subjective assessment of knowledge acquisition and behaviour was assessed through anonymised Likert scale questions administered before and after course completion (appendix 2) and included assessment of team working skills, learner reaction and reflection on the time they had spent preparing for each session.

### Statistical Analysis

Attendance at each session varied according to the doctor’s rota and leave commitments. Team continuity was assessed based on the number of team members present per session in relation to the total number or pool of doctors for that team. An overall group continuity score was calculated using the mean continuity score over the four sessions.

Data from iRAT and tRAT scores was entered into Microsoft Excel for analysis. Each team received an average iRAT score for each session (total iRAT score/no. participants in team) in addition to a minimum and maximum iRAT score for each individual team member. Teams obtained a total tRAT score and ‘1st choice tRAT score’, taken as the team’s score if only the first answer counted—to enable better comparison against iRAT scoring. Question difficulty was assessed by analysing average iRAT and tRAT scores for each question.

Pre- and post-course quantitative feedback for learner reaction, knowledge and behaviour were compared using unpaired *t* tests. Pearson correlation coefficients were calculated to identify influencing factors. Qualitative feedback for each session was transcribed, analysed and categorised into themes to identify overall reaction to TBL.

## Results

### Team Composition and Continuity

Of the population of 48 first year postgraduate doctors all received at least 1 session delivered using TBL methodology. Average attendance was 2.5/4 sessions (range 1–4). Six teams were originally created, reducing to 5 teams with an average team membership of 5.85 doctors per session taken from a pool of 9.6 (8–11) team members. Teams always had between 5 and 7 members, except for one group which had 4 members on one occasion. Continuity within the teams varied significantly ranging from 40 to 88% (where 100% equals all potential members of a team being present at the same session). Across the four sessions overall team continuity was 65%.

### Learner Reaction to TBL

After completion of the course, trainees rated ‘enjoyment of the delivery of the transfusion curriculum through TBL’ as 7.1/10 (3–10, *n* = 27). There was no correlation between enjoyment and the number of sessions attended, nor with subjective knowledge in transfusion at the end of the course. There was a strong positive correlation between enjoyment and reported actual time spent in preparation for the sessions (*r*^2^ 0.40).

Qualitative feedback was predominantly positive. Aspects enjoyed by the trainees included the interactive nature of TBL, learning from each other, engaging with real life clinical scenarios and working in a team. The recurring themes within the negative comments concerned either a lack of time to prepare for the session or the negative impact of lack of continuity within teams. When asked directly if ‘TBL had ensured efficient use of their time’, trainees rated this slightly lower than other areas at 6.8/10 (range 3–10). There was a strong positive correlation between this variable and time spent in preparation for sessions (*r*^2^ = 0.5), indicating that those who spent longest preparing found it most time efficient.

### Knowledge Acquisition

Objectively trainees demonstrated increased knowledge working as a team; reflected by an increase in the tRAT score over the mean individual iRAT score for each session and overall (Table 1), which held true in the majority of cases when considering only the first tRAT answer. The tRAT score exceeded the maximum iRAT score per team in 90% cases.

**Table 1:** With TBL, learners demonstrate increased knowledge acquisition working as a team rather than individually. Results show mean (range) individual readiness assurance test (iRAT) score according to team and session; team readiness assurance test (tRAT) scores when reviewing first answer only (tRAT 1st answer) and overall team score (tRAT overall). Awarded 10 points for correct answer 1st time, 6 for 2nd, 2 for 3rd and 1 for 4th. Maximum score for each is 100

Session	TBL orientation			1: Indications for transfusion			2: Transfusion reactions			3: Clotting and haemostasis		
Team	iRAT average	tRAT 1st answer	tRAT overall	iRAT average	tRAT 1st answer	tRAT overall	iRAT average	tRAT 1st answer	tRAT overall	iRAT average	tRAT 1st answer	tRAT overall
1	26 (10–40)	60	63	42 (30–50)	60	76	41.4 (10–80)	50**	67**	37 (10–50)	50	70
2	32 (20–50)	50	55	42 (20–80)	70**	83	32 (10–50)	30*/**	55	32.9 (20–40)	50	66
3	22.5 (10–30)	30	67	n/a	n/a	n/a	n/a	n/a	n/a	n/a	n/a	n/a
4	30 (20–40)	40	64	48.3 (10–90)	90	92	33.3 (20–50)	30*/**	44**	28.3 (20–50)	50	55
5	18.3 (10–30)	40	54	42 (40–50)	60	76	47.1 (30–70)	60**	75	28.3 (30–40)	20*/**	64
6	24 (10–30)	40	64	31.7 (0–50)	50	69	30 (20–40)	30*/**	56	30 (10–40)	50	67
Total population	25 (10–50) *n* = 29	43	61	41 (0–90) *n* = 27	66	79	37 (10–80) *n* = 32	40	59	32 (10–50) *n* = 29	44	66

*Average iRAT > tRAT 1st answer. **Max iRAT score > tRAT score. Results suggest a problem with the delivery or assessment of session 2 - see discussion

Comparison of trainee confidence levels pre- and post- the TBL transfusion course showed a significant fall concerning prescribing the right blood products, for the right patient, at the right time and significant increase in confidence level dealing with Acute Transfusion Reactions (Table 2). Both had a negative correlation with perceived time spent in preparing for sessions; the longer a trainee felt they had spent in self-study, the lower they were likely to rate themselves as confident prescribing blood products (*r*^2^ = − 0.4) or dealing with ATRs (*r*^2^ = − 0.6). Confidence in these areas was not correlated with the number of transfusion sessions attended. When asked directly whether TBL had increased their knowledge of transfusion medicine, there was a positive response of 7.3 (scale 1–10, range 4–10, *n* = 27). Subjective increase in knowledge had a strong positive correlation with reported actual time spent in preparation for sessions (*r*^2^ = 0.47).

**Table 2:** Subjective assessment of knowledge and behaviour concerning transfusion medicine both before (*n* = 28) and after (*n* = 27) completion of between 1 and 4 TBL sessions covering the transfusion curriculum

On a scale of 1–10, where 1 is not at all confident and 10 is totally confident, please circle the number which best represents...	Pre	Post
How confident you are that you always prescribe the right blood products, for the right patient, at the right time?	8.2	7.2*
How confident you are dealing with Acute Transfusion Reactions (ATRs)	5.4	6.7*
How often are you in agreement with the transfusion decisions made by your seniors?	6.3	7.0
How likely would you be to challenge a decision made by your senior concerning transfusion?	5.1	5.9

*p < 0.05

### Change in Behaviour

Subjectively there was no significant change in behaviour concerning agreement with transfusion decisions or likelihood of challenging a senior’s decision (Table 2). Trainees did feel ‘TBL had improved their ability to make clinical decisions concerning transfusion practice’, rating it positively at 7.3 (scale 1–10, range 4–10, *n* = 27). There was a strong positive correlation between perceived ‘ability to make clinical transfusion decisions’ and reported time spent on preparation for sessions (*r*^2^ = 0.43). Trainees felt TBL had improved their team working skills, scoring it 7.0 (scale 1–10, range 3–9, *n* = 27).

### Peer Evaluation

Peer evaluation forms were completed by 31 trainees. Quantitative feedback varied by only 1 point (e.g. 4 points to everyone in group with 5 to a single individual) and the team member scoring 5 was rarely the same person. One team had an obvious outlier who did not contribute to the team, scoring 1–2 points from each other team member. This individual’s own reflection on their input was ranked as adequate/appropriate for all areas although of note they had only filled in part of the peer evaluation from and had included no free text.

Free text was included in 74% of peer evaluation forms. Analysis showed a tendency to include comments concerning ‘Things I appreciate about this team member’, e.g. thoughtful input, well prepared, good contributions. Fewer comments were made regarding ‘Things I would like to request of each team member’, with most trainees stating ‘nothing’, or ‘no concerns’. Infrequently potentially useful comments were included, e.g. ‘be more vocal’ or ‘have more confidence in answering questions’. Trainees that these comments referred to always felt their contribution to the group discussion was adequate.

### Reported Preparation

Learner preparation for sessions was rated in two places; within the peer evaluation where names were recorded, and within anonymous feedback. Learner reported preparation varied widely between these two methods. In anonymous evaluation (*n* = 27) preparation time was most often reported as 30–59 min (9/27), varying from none (5/27) to 1–2 h (4/27). Preparation was described as adequate in 56% and excellent in 30% of this anonymous feedback, however, within the named peer evaluation (*n* = 30), preparation was described as excellent in only 3% and absent (1/30) or lacking (20/30) by 70% of doctors.

## Discussion

Trainees attended an average of 2.5 out of 4 ‘compulsory’ TBL sessions due to on-call commitments or annual leave. Overall team continuity, represented by the number of team members present per teaching session as a proportion of the total number (or pool) of doctors for that team, was only 65% across the 4 sessions due to these on-call/leave absences. Group size within TBL is recommended to be 5–7 as fewer members provide inadequate assets, e.g. knowledge to tackle complex problems [15, 17]. Despite poor team continuity, appropriate group size was ensured for all teams except on one occasion. Team dynamics, for example communication skills and perceived self-learning, are improved by TBL [18]. Doctors should be used to forming ad hoc teams as part of their professional role; however, qualitative feedback in this postgraduate cohort demonstrates a negative impact on learning from poor team continuity. The authors therefore propose removing continuity as a potential issue, by condensing the TBL format into a single session when using it in the postgraduate medical setting.

Evaluating reactions involves assessing how trainees (and other stakeholders) respond to the introduction of TBL and is achieved subjectively using feedback or evaluation forms. Quantitative data is easier to use for comparison although qualitative evaluation is often more useful to identify what works (or doesn’t) and for what reasons [19]. Indirect methods may form an alternative quantitative approach to objectively evaluate the acceptability of TBL to learners. As an example, in Oman TBL has demonstrably improved attendance at mandatory undergraduate teaching, demonstrating the increased interest the methodology provokes [20]. This method of evaluation is, however, not applicable in this setting, where attendance was compulsory unless on call or annual leave.

In this study both quantitative and qualitative feedback by doctors was positive. Incorporating doctors’ evaluation of TBL into implementation is of importance as the first systematic review of TBL in the health profession setting showed mixed learner reaction [21]. Some researchers report preference for TBL and others for the alternative method, particularly small group learning, although few meet statistical significance. Negative reactions to TBL may result from increased workload necessary pre-session, alongside new emphasis on peer assessment and accountability, and precautions to mitigate these potentially negative reactions to TBL should be taken [21]. In our study, the recurring themes within the negative qualitative comments concerned either a lack of time to prepare for the session or the negative impact of lack of continuity within teams. Additional comments included a dislike for the time TBL takes, the need to speak in front of the whole group, too much group work, lack of ‘teaching’ and a request for more clarification at the end of each session. The latter can be easily addressed through the creation of post-session handouts that draw together key learning outcomes and future learning resources.

Reaction to an educational method is thought to be fundamentally linked to learner response to learning and learner behaviour, however of interest in this study, we found no correlation between learner enjoyment and subjective knowledge acquisition, or learner enjoyment and number of sessions attended. The authors propose the former may reflect the huge enjoyment some learners get from the scratch-card and/or social element of TBL sessions, combined with some learners subjectively gaining most from the individual pre-course material and not enjoying the interactive elements of the session. The authors feel the latter is because TBL is a bit like Marmite; you either love it or hate it, it doesn’t grow on you. These findings and the impact of team continuity in TBL within the postgraduate/graduate medical setting could be better understood through future focus group work in this cohort.

Overall, the authors speculate that this mixed learner reaction is more likely a result of unmet expectations, as in contrast to traditional small group learning, it is through personal preparation and group discussion that trainees gain knowledge in TBL. The main role of the tutor (and faculty) is to set appropriate preparatory material, assurance tests and application scenarios to facilitate this learning process, with the relatively brief whole group discussions serving only to clarify misunderstandings and expand on areas of controversy. The Foundation curriculum clearly states that postgraduate doctors have a professional and personal responsibility for their own learning [16]. It was proposed that by ensuring the one-off TBL orientation session clearly explained the role expected of both trainee and tutor, that these potentially negative reactions could be ameliorated. The attendance at orientation was however only 60%, which may explain why these views persisted in feedback, and again supports the need for qualitative research in the postgraduate/graduate medical setting to better understand learner reaction.

Successfully combining trainee assessment within educational delivery requires adequate engagement of the trainees with the preparatory work. We found a positive correlation between reported time spent in preparation for TBL sessions and both enjoyment and subjective increase in knowledge. There was a negative correlation between reported time spent preparing and clinical confidence levels post-TBL course; suggesting that the more trainees knew, or realised they didn’t know, the less likely they were to feel clinically confident. The significance of subjective clinical confidence is however questionable as confidence is not related to knowledge, although confidence levels may affect behaviour [5]. Variability in engagement with preparatory work is bound to happen due to the different styles of learning engagement and because the TBL course was delivered as part of mandatory training rather than a course chosen by the doctor. Awarding an overall grade for the course, combining iRAT, tRAT, tAPP and peer evaluation scores, can provide incentive to motivate learners in their degree of preparation reading although in this case we provided incentives from their tRAT scores only.

Trainee-reported preparation time was strikingly less when doctors had to document their name on feedback forms in comparison to anonymised responses. It may be that when anonymised, doctors are overestimating their preparation as they may not know how much preparation is expected or may be trying to please faculty. More likely in named responses, doctors are under reporting their preparation, with the mentality that by playing down their commitment, they are inflating their innate competency. Qualitative feedback suggests that engagement with preparatory material in this population is potentially sub-optimal, although in this cohort of postgraduate doctors, they do have the benefit of clinical experience to supplement their pre-course reading and team contribution.

Faculty reaction to the introduction of TBL as a new teaching method was positive within the small number of individuals involved, as per other studies, although creation of the TBL sessions was noted to be considerably more time consuming than using ‘traditional’ methods [22]. Evaluation of wider stakeholders was not applicable in this setting. There is speculation that the apparent aversion to TBL in the UK may stem from a lack of familiarity with the technique, a conviction to stick with problem-based learning (PBL) having designated so many resources to developing this technique and/or a teacher bias towards classical lectures [9]. We would tend to agree, as introduction of TBL requires unique resource materials and an understanding of the theory and delivery strategies, although we hope that with the recent increased emphasis placed on the theory of education in the postgraduate medical setting, we may find a gradual move towards this and more innovative methods of education delivery.

The role of learner assessment is critically interwoven into the functioning of TBL and in doing so forms part of evaluation. RAT with immediate feedback provides both formative and summative assessment of learning to learners and tutors on how well they have understood the base knowledge. It also complies with the basic requirements of assessment as set out by the World Federation for Medical Education [23]. TBL has been shown to result in improved (or neutral) knowledge acquisition although studies are often flawed by non-concurrent group analysis and cohort designs [20]. We found team knowledge improved over individual knowledge with tRAT scores exceeding team member’s individual iRAT scores in 90% of cases. Where team knowledge did not exceed individual knowledge, this is more likely to reflect deficiencies in the session or assessment material used, as supported by a lower presentation score for the session on ATRs (Table 3), where this was observed [24]. Because of the success of cooperative learning, there is an expectation that only 4% of iRAT scores will be higher than the lowest tRAT score [25]. We found this to be the case in 9% although again this suggests a problem in the design of the ATR session, where this was observed in 8/32 cases, in comparison to 2/85 in the rest of the course.

**Table 3:** Anonymous session feedback provided by trainees to the education coordinator at the end of each TBL session. Range 1–5 with 3 = average, 4 = very good, 5 = excellent

	TBL orientation	Indications for transfusion	Acute transfsuion reactions (ATR)	Haemostasis and thrombosis
Relevance	3.7	4.3	4.2	4.2
Content	3.7	4.3	4.1	4.2
Presentation	3.8	4.2	3.9	4.2

Within TBL, evaluating behaviour and evaluating learning are entwined. Behaviour determines engagement with both the preparatory material and within the team and hence dictates the degree of learning that takes place. Assessing how trainees respond to clinical situations and how they apply their knowledge forms another part of behaviour evaluation. This is particularly important in transfusion medicine as doctors are required to assimilate clinical findings, laboratory results and make risk assessments to reach the most appropriate management plan. Team application scenarios within the TBL process provide trainees with authentic clinical scenarios to apply their knowledge and debate their decision-making choices. In similarity to the Readiness Assurance Tests, this assessment of behaviour provides both summative and formative evaluation for stakeholders, with assessment of learning and assessment for learning. In this study, the faculty decided to use free text answers for tAPP scenarios to make cases truer to clinical practice, however, this meant scoring was logistically more difficult to apply and change in behaviour was evaluated by subjective reporting rather than objective practice in tAPP cases, which may have been more representative.

Additional behavioural attributes can be assessed to elicit the effectiveness of TBL as an educational tool, e.g. communication and team working skills as part of the peer evaluation process, with TBL previously being shown to improve team emotional intelligence [26]. In our study population, we found satisfactory engagement with the peer evaluation process, with 74% documenting qualitative comments. The quantitative scoring system appeared less helpful as awarded marks were very similar. Engagement with peer evaluation will have likely been influenced by the close-knit relationship between most trainees within the group (not wanting to upset anyone) and by the poor team continuity.

Ideally education and learning should be directly linked to improvements in patient care and health outcomes, which would correlate with Kirkpatrick’s ‘results’ level of learning outcome [27]. Attempting to evaluate the effect of TBL on transfusion safety, particularly after limited application to one set of health personnel, is impractical because errors are relatively rare and often occur at multiple levels. In addition, our cohort of doctors may not be the ones making decisions to transfuse but rather be carrying out instructions from seniors, a discrepancy that has been highlighted previously when evaluating whether to focus educational interventions on attending clinicians or residents [28]. It must also be remembered that demonstrating improved results following an educational intervention does not necessarily mean durable results. A prior systematic review of transfusion interventions identified a reduction in utilisation of blood immediately after the introduction of interventions, yet this returned to baseline as soon as the intervention was over or when the same interventions were still ongoing [29].

From this research, it can be concluded that implementation of stand-alone TBL sessions in transfusion medicine would be an effective and clinically relevant method of delivering postgraduate medical education in the United Kingdom, although current familiarity with this tool may limit its application. Implementation of serial TBL sessions in the postgraduate setting is hindered by irregular attendance even at ‘compulsory’ teaching, which results in poor team continuity and negatively impacts on the principles of cooperative learning that govern TBL. As the formation of ad hoc teams is the norm in clinical practice, further qualitative research into the impact of team continuity in the postgraduate setting is recommended, and methods to address the impact of poor team continuity should be addressed in session design where serial TBL sessions are planned. Overall, both trainee and faculty reaction to TBL was positive, particularly when the learners engage adequately with preparatory material. Doctors demonstrated increased knowledge acquisition through TBL in addition to the development of auxiliary skills, e.g. team working. Delivery of the transfusion curriculum to postgraduate doctors through TBL more accurately reflects the clinical application of knowledge and skills they require for safe transfusion practice and is an effective educational method for this purpose.
